# Au_36_(SR)_22_ Nanocluster and a Periodic Pattern from Six to Fourteen Free Electrons in Core Size Evolution

**DOI:** 10.1021/jacsau.4c00152

**Published:** 2024-04-16

**Authors:** Yitong Wang, Christopher G. Gianopoulos, Zhongyu Liu, Kristin Kirschbaum, Dominic Alfonso, Douglas R. Kauffman, Rongchao Jin

**Affiliations:** † Department of Chemistry, Carnegie Mellon University, Pittsburgh, Pennsylvania 15213, United States; ‡ Department of Chemistry and Biochemistry, University of Toledo, Toledo, Ohio 43606, United States; § National Energy Technology Laboratory, United States Department of Energy, Pittsburgh, Pennsylvania 15236, United States

**Keywords:** atomically precise nanoclusters, Au_36_(SR)_22_, optical properties, electronic structure, structural evolution

## Abstract

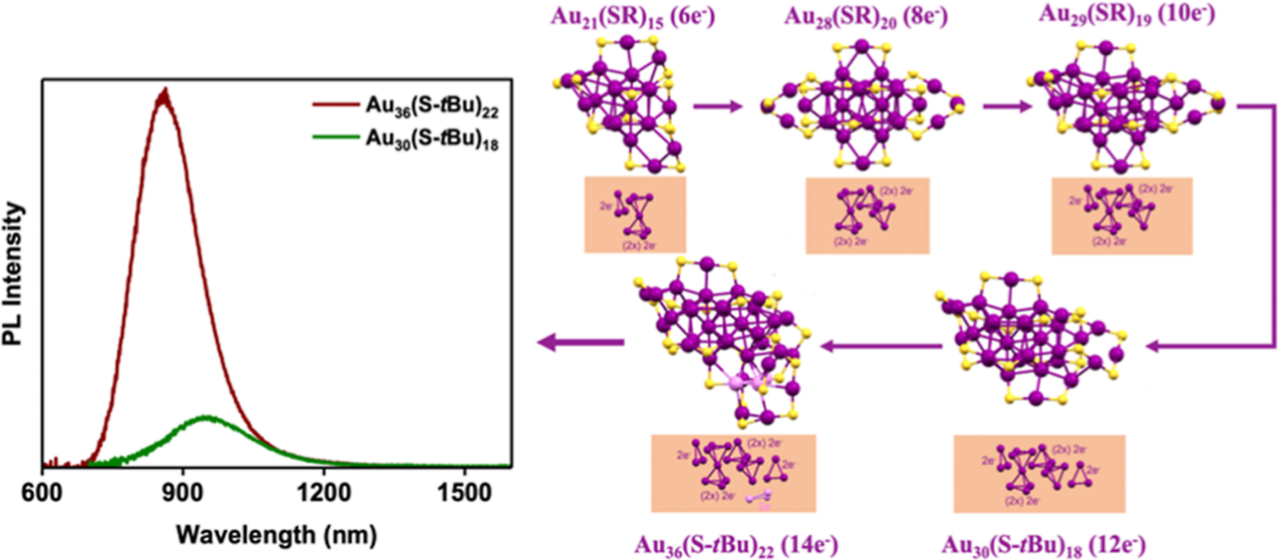

An Au_36_(S-*t*Bu)_22_ nanocluster (NC) is synthesized using the bulky *tert*-butyl thiol as the ligand. Single-crystal X-ray crystallography reveals that it has an Au_25_ core which evolves from the Au_22_ core in the previously reported Au_30_(S-*t*Bu)_18_, and the Au_25_ core is protected by longer staple-like surface motifs. The new Au_36_ NC extends the members of the face-centered cubic structural evolution by adding an Au_3_ triangle and an Au_4_ tetrahedron unit. Additionally, it is found that Au_36_ emits near-infrared photoluminescence at 863 nm with a quantum yield (QY) of 4.3%, which is five times larger than that of Au_30_(S-*t*Bu)_18_—the closest neighbor in the structural evolution pattern. The higher QY of Au_36_ is attributed to a larger radiative relaxation (*k*_r_), resulting from the structural effect. Finally, we find that the longer staple motifs lead to enhanced stability of Au_36_(S-*t*Bu)_22_ relative to Au_30_(S-*t*Bu)_18_.

## Introduction

Atomically precise gold nanoclusters (NCs) exhibit distinct photoluminescence (PL) and catalytic properties owing to their ultrasmall size, excellent stability, and unique core/staple-shell structure.^1−8^ Recent studies based on X-ray crystallography have established some structure–property correlations,^3−6^ revealed insights into the stability^7^ and physicochemical properties^8^ of the NCs, and even aided in predicting some unknown NCs and configurations.^9−14^ However, there has been less understanding of the structural evolution from smaller to larger sizes for a specific core packing mode.^15^ Unlike plasmonic nanocrystals of gold, which generally adopt the face-centered cubic (fcc) structure,^16^ gold NCs below the size of Au_279_(SR)_84_ (2.25 nm core diameter^17^) have been found to pack their core atoms into various modes, including the fcc,^13,14,17^ hexagonal close-packed (hcp),^15^ body-centered cubic (bcc),^18^ and polyhedron-based cores such as M_4_ tetrahedron, M_7_ decahedron, and M_13_ icosahedron.^7,18^ For the Au NCs with fcc-structured cores, two different evolutionary patterns have been discovered^19−21^ and discussed below.

Using aromatic ligands, Zeng *et al.* discovered in 2016 a series of Au NCs with the cores formed from successively adding tetrahedral Au_4_ units.^19^ The number of Au atoms and ligands follows a common formula, Au_8*n*+4_(TBBT)_4*n*+8_ with *n* ranging from 2 to 6, which gives rise to Au_20_(TBBT)_16_, Au_28_(TBBT)_20_, Au_36_(TBBT)_24_, Au_44_(TBBT)_28_, and Au_52_(TBBT)_32_ (TBBT; *tert*-butylbenzene thiol). The number of free valence electrons can be calculated by means of the grand unified model (GUM).^22^ In this model, the gold core is viewed as a fusion or combination of triangular Au_3_ and tetrahedral Au_4_ elementary blocks with each contributing two free electrons to the entire core. In this context, the tetrahedral Au_4_ units in the cores of the Au_8*n*+4_(TBBT)_4*n*+8_ series evolve in a double-helical pattern along the [001] direction.^13,14,19^ The number of free electrons periodically increases from 4e^–^ (accounting for the two Au_4_ units) in Au_20_(TBBT)_16_ to 8e^–^ (4 units) in Au_28_(TBBT)_20_, 12e^–^ (6 units) in Au_36_(TBBT)_24_, 16e^–^ (8 units) in Au_44_(TBBT)_28_, and 20e^–^ (10 units) in Au_52_(TBBT)_32_.

On the other hand, by employing bulky nonaromatic ligands (*e.g.*, *tert*-butyl thiolate, S-*t*Bu), Dass and Zheng groups^20,23^ reported a dimeric fcc Au_30_(S-*t*Bu)_18_, and Pei’s group summarized a second pattern of fcc structure evolution implicated by the Au_21_(S-*t*Bu)_15_ structure,^24^ where the core is built up by adding the joint cuboctahedron Au_13_ units. The core of the dimeric fcc Au_30_(S-*t*Bu)_18_ can be viewed as an Au_20_ bicuboctahedron formed by fusion of two Au_13_ cuboctahedra, where six of the Au atoms are shared. Based on the GUM model,^22,25^ two triangular Au_3_ units and four tetrahedral Au_4_ units can be identified in Au_30_(S-*t*Bu)_18_,^24^ which contribute 12 free electrons to the core. For Au_21_(S-*t*Bu)_15_, its Au_10_ core contains six free electrons that account for one bitetrahedral Au_7_ unit (two tetrahedral Au_4_ units) and one triangular Au_3_ unit.^24^ In subsequent work, Li *et al.*(26) further found a heterodimeric Au_29_(S-Adm)_19_ that was earlier predicted by Pei *et al.*,^10^ which bridged up the second fcc growth pattern along with the earlier reported Au_28_(S-*c*-C_6_H_11_)_20_ and Au_30_(SR)_18_ (SR = S-Adm or S-*t*Bu).^24^

Recently, Liao *et al.* reported an evolutionary case for the first fcc series^13,14,19^ discussed above, namely, Au_56_(TBBT)_34_.^27^ It contains 22 free electrons and is constructed by adding four gold atoms onto Au_52_(TBBT)_32_ along its [001] direction based upon X-ray crystallographic analysis. However, new crystal structures that extend the second fcc evolutionary pattern are still desirable.

In this work, we serendipitously discovered a new fcc-structured Au NC, namely, Au_36_(S-*t*Bu)_22_, by using bulky S-*t*Bu as the ligand. This NC belongs to the second fcc series, and its gold core evolves by an additional triangle Au_3_ on the side of the core of Au_30_(S-*t*Bu)_18_. The optical spectroscopy analysis and time-dependent density functional theory (TDDFT) simulations reveal that the additional two free electrons in the core lead to a dramatic change in the optical and electronic properties of this fcc dimeric NC. Furthermore, it is found that the new Au_36_(S-*t*Bu)_22_ exhibits NIR emission with a 4.3% quantum yield (QY) in dichloromethane (DCM) at room temperature, which is much higher than that of Au_30_(S-*t*Bu)_32_ (0.8%). The thermal and photostability of Au_36_(S-*t*Bu)_22_ and Au_30_(S-*t*Bu)_18_ are further investigated. Such materials hold potential in various applications such as photo- and electro-catalysis,^1−4^ bioimaging,^5^ and optoelectronics.^7^

## Results and Discussion

### Synthesis and Optical Properties of Nanoclusters

Details of the synthesis of Au_36_(S-*t*Bu)_22_ are provided in the Supporting Information. Briefly, HAuCl_4_·3H_2_O was dissolved in THF, followed by adding *tert*-butyl thiol. After 30 min, Au^I^–S-*t*Bu was reduced by NaBH_4_. The reaction was allowed to proceed for 3 h and then the solution was evaporated to dryness. The crude product was washed thoroughly with methanol, extracted by DCM, redissolved in toluene, and stored at room temperature for incubation. After 1 week, the mixture was separated by preparative thin-layer chromatography (Figure S1). The Au_36_(S-*t*Bu)_22_ NC was crystallized by vapor diffusion of methanol into a toluene solution of the NC.

**Figure 1:**
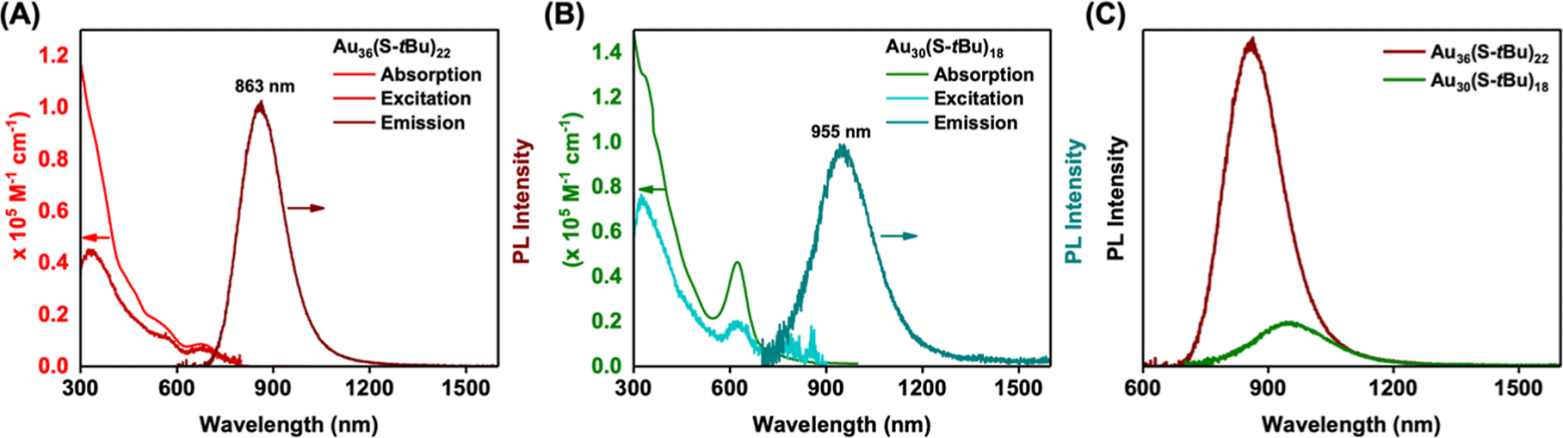
(A,B) UV–vis absorption spectra, normalized PL spectra, and normalized PLE spectra of Au_36_(S-*t*Bu)_22_ and Au_30_(S-*t*Bu)_18_ in DCM under ambient conditions (note: slit width for PL and excitation measurements: 8 nm). (C) Comparison of PL intensities for Au_36_(S-*t*Bu)_22_ and Au_30_(S-*t*Bu)_18_ in DCM under ambient conditions [the same optical density (OD), 0.1 OD, at the excitation wavelength of 365 nm].

The UV–vis spectrum of Au_36_(S-*t*Bu)_22_ exhibits a peak at 677 nm and less prominent ones at 570, 524, 469, 445, and 380 nm (Figure 1A, red). The absorption coefficient at 677 nm is ε = 8.6 × 10^3^ M^–1^ cm^–1^, which is typical of Au_*n*_(SR)_*m*_ NCs. We found that Au_36_(S-*t*Bu)_22_ emitted PL in the near-infrared (NIR) region (peak at ∼863 nm) (Figure 1A), which is red-shifted relative to the previously reported PL of Au_36_(TBBT)_24_ that has the same number of gold atoms (peak at 770 nm).^28^ We also compared with the smaller Au_30_(S-*t*Bu)_18_, which exhibits a sharp absorption peak at 620 nm (ε = 4.6 × 10^3^ M^–1^ cm^–1^) and emission at 955 nm, Figure 1B. The PLQY of Au_36_(S-*t*Bu)_22_ was determined to be 4.3% by a relative method, which is about five times greater than that of Au_30_(S-*t*Bu)_18_ (PLQY: 0.8%) (Figure 1B). For both NCs, their PL excitation spectra track the absorption profiles, indicating that the PL comes from the NCs (as opposed to impurities). The PL intensities of the two NCs are compared in Figure 1C.

**Figure 2:**
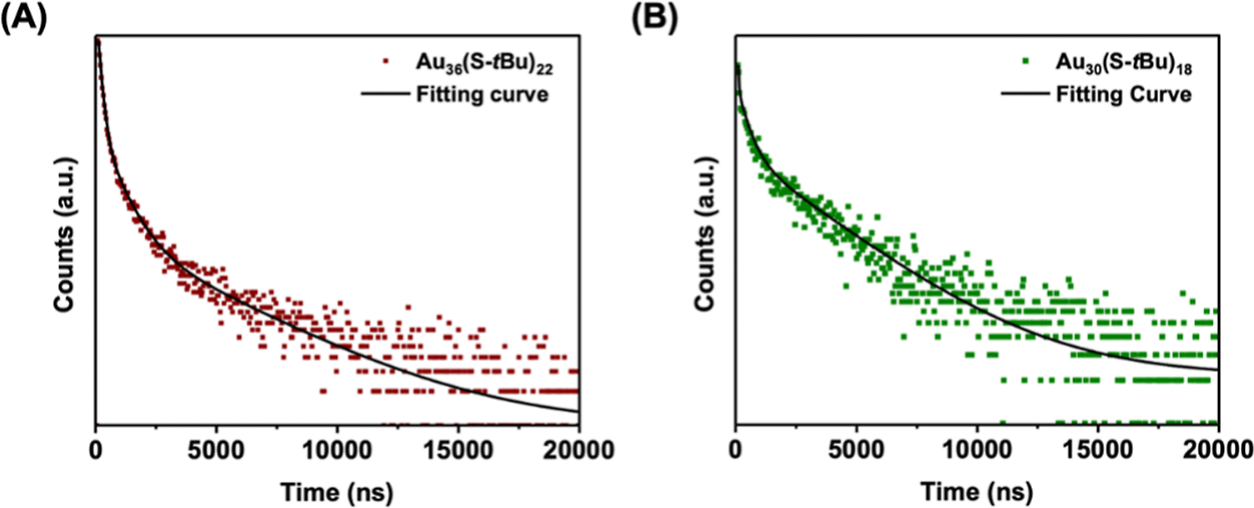
PL decay profiles of (A) Au_30_(S-*t*Bu)_18_ and (B) Au_36_(S-*t*Bu)_22_ in a dilute toluene solution.

The PL lifetimes of Au_36_(S-*t*Bu)_22_ and Au_30_(S-*t*Bu)_18_ in toluene solutions under ambient conditions were measured by a time-correlated single photon counting technique (see details in the Supporting Information). Data fitting by exponential functions identified three lifetime components in Au_36_(S-*t*Bu)_22_, with τ_1_ = 145 ns (amplitude *A*_1_ = 46%), τ_2_ = 740 ns (*A*_2_ = 31%), and τ_3_ = 3941 ns (*A*_2_ = 23%) (Figure 2A), and the average lifetime (τ_av_ = 1200 ns) was calculated by the formula
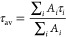
1where *A*_*i*_ is the relative amplitude and τ_*i*_ is the lifetime component. Similarly, we found τ_1_ = 23.7 ns, τ_2_ = 437 ns, and τ_3_ = 3427 ns (τ_av_ = 2747 ns) for Au_30_(S-*t*Bu)_18_ (Figure 2B). The photophysical properties are summarized in Table 1. The significantly higher radiative rate constant *k*_r_ of Au_36_(S-*t*Bu)_22_, *i.e.*, more than 10 times that of Au_30_(S-*t*Bu)_18_, explains the much stronger PL of Au_36_(S-*t*Bu)_22_ than that of Au_30_(S-*t*Bu)_18_.

**Table 1:** Photophysical Data of Au_30_(S-*t*Bu)_18_ and Au_36_(S-*t*Bu)_22_ in Toluene under Ambient Conditions^a^

solution phase	Au_36_(S-*t*Bu)_22_	Au_30_(S-*t*Bu)_18_
Φ_PL_ (%)	4.3	0.8
τ_av_ (ns)	1200	2747
*k*_r_ (s^–1^)^b^	3.6 × 10^4^	2.9 × 10^3^
*k*_nr_ (s^–1^)^c^	8.0 × 10^5^	3.6 × 10^5^
*E*_g_ (eV)	1.52	1.45
EE (eV)	1.44	1.30

a Φ_PL_: quantum yield, *k*_r_: radiative rate constant, *k*_nr_ nonradiative rate constant, *E*_g_: optical gap energy (taken at the absorbance onset in the spectrum), and EE: emission energy at the peak.

b Calculated by *k*_r_ = Φ_PL_·τ_av_^–1^.

c Calculated by *k*_nr_ = (1 – Φ_PL_)·τ_av_^–1^.

### X-ray Structures of Nanoclusters

**Figure 3:**
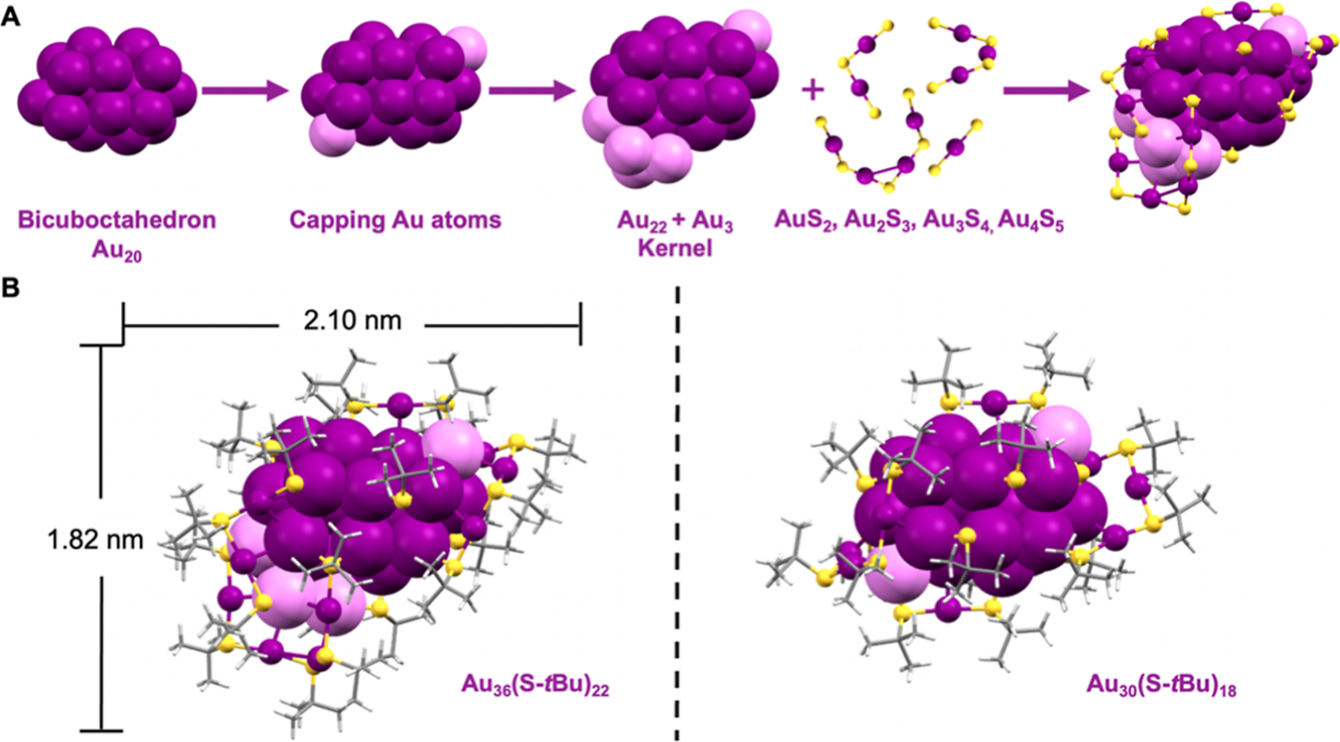
(A) Structural anatomy of Au_36_(S-*t*Bu)_22_. (B) Comparison of the X-ray structures of Au_30_(S-*t*Bu)_18_ and Au_36_(S-*t*Bu)_22_. Color code: purple and magenta = Au; yellow = S; gray = C; and white = H.

To obtain structural insights, we crystallized the Au_36_(S-*t*Bu)_22_ NC by a vapor diffusion method. The Au_36_(S-*t*Bu)_22_ NC crystallized in the triclinic space group *P*1̅ (Figure S2 and Table S1 in the Supporting Information). The X-ray structure shows an innermost core of fcc-Au_20_ bicuboctahedron (Figure 3A), which is the same as the Au_20_ core previously discovered in Au_28_(SR)_20_.^19^ Capping two Au atoms onto the two ends of Au_20_ gives rise to a prolate Au_22_ core, which corresponds to the same core in the previously reported Au_30_(SR)_18_.^20^ Following the fcc-type core evolutionary pathway, a triangle-shaped Au_3_ unit is attached to the bottom of prolate Au_22_, resulting in an asymmetric Au_25_ core. Compared with the Au_20_ core of fcc-Au_28_(SR)_20_ and the Au_22_ core of fcc-Au_30_(SR)_18_, the presence of an additional Au_3_ unit in the Au_25_ core of Au_36_(S-*t*Bu)_22_ significantly modifies the surface structure. As a result, the rest of the Au atoms participate in a variety of staple motifs in order to protect the Au_25_ core. Four different types of staple motifs can be identified, namely, Au(SR)_2_, Au_2_(SR)_3_, Au_3_(SR)_4_, and Au_4_(SR)_5_ staples (Figure 3A). Interestingly, the surface of the Au_22_ portion of the Au_25_ core of Au_36_(S-*t*Bu)_22_ shows the same type and arrangement of surface staple motifs that exist in fcc-Au_30_(SR)_18_ (Figure 3B, right). One of the two face-capping Au atoms on one side of the Au_22_ portion acts as a linker between the Au_3_(SR)_4_ and Au(SR)_2_ staples. However, the staple arrangement in Au_30_(SR)_18_ is not sufficient to accommodate the additional Au_3_ unit on the other side of the Au_22_ portion; thus, Au_2_(SR)_3_ and Au_4_(SR)_5_ are formed to protect the surface.

Here, we discuss the structural effects on the PL properties. Previously, Zhuang *et al.* reported PLQY of Au_44_(TBBT)_28_ and Au_42_(TBBT)_26_ and suggested that an increase in the number of core gold atoms and the staples enhances the PL in Au_44_(TBBT)_28_.^29^ On the other hand, Zhou *et al.* demonstrated that the addition of two core atoms in Au_48_(*m*-MBT)_26_*versus* Au_46_(*m*-MBT)_26_ reduces the overall PLQY of Au_48_(*m*-MBT)_26_ (where, *m*-MBT = *meta*-methylbenzenethiolate).^30^ Furthermore, they found that the more surface motifs [four Au_3_(SR)_4_ staples in Au_28_(CHT)_20_-II] than the isomeric Au_28_(CHT)_20_-I [the latter bearing only two Au_3_(SR)_4_ staples] increase the surface rigidity, hence, leading to an overall 7.4-fold PLQY enhancement in Au_28_(CHT)_20_-II compared to the Au_28_(CHT)_20_-I isomer (where, CHT = cyclohexanethiolate).^31^ In our current study, the more emissive Au_36_(S-*t*Bu)_22_ has three more gold atoms in the core than that of Au_30_(S-*t*Bu)_18_, which leads to an enhancement in the *k*_r_ according to the Einstein transition probability of spontaneous emission (Table 1).^4,32,33^

**Figure 4:**
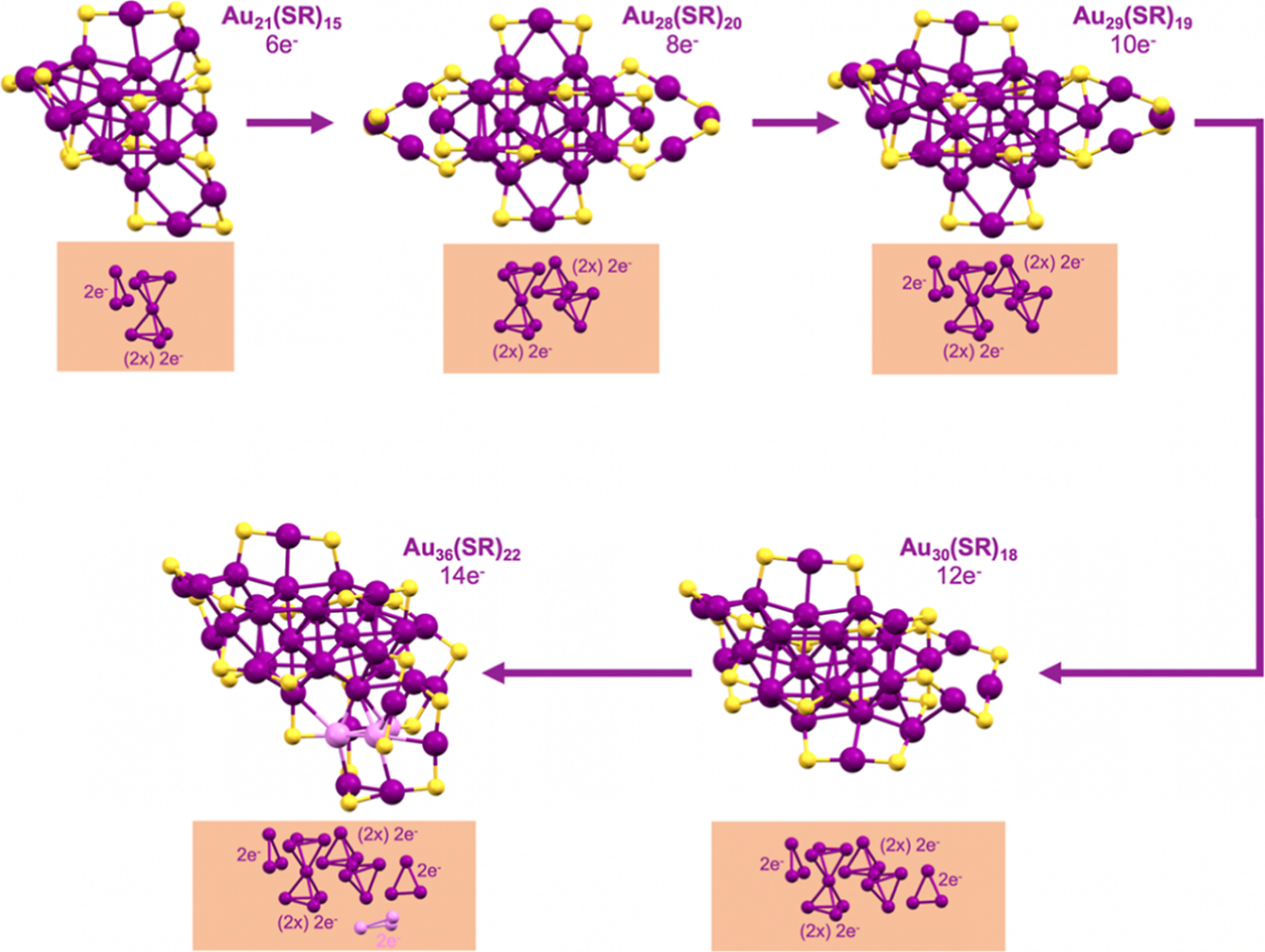
Structural evolution of fcc Au cores based on the GUM. Color code: purple, magenta = gold; yellow = sulfur; and R groups are not shown for clarity.

The established fcc-core evolutionary pathway^24^ based on the joint cuboctahedral units can be further extended by the discovery of Au_36_(S-*t*Bu)_22_, as depicted in Figure 4. In previous work, Au_21_(S-*t*Bu)_15_ was found to adopt a complete monocuboctahedron core by analyzing the Au–Au bond length, which can be further divided into an Au_7_ bitetrahedral unit and an Au_3_ triangle unit.^24^ When an Au_4_ tetrahedral unit is added to the Au_10_ core, an Au_14_ core is formed, which features a fusion of cuboctahedral units. This core can be found in Au_28_(TBBT)_20_,^34^ Au_28_(CHT)_20_-I, and Au_28_(CHT)_20_-II;^31^ note that the latter two are isomeric structures reported by Wu’s group. Given the large difference in the ligands (aromatic TBBT *vs* nonaromatic CHT), the only difference in these three Au_28_(SR)_20_ NCs is the arrangement of the remaining “Au_14_(SR)_20_” unit for the gold–sulfur interface, which demonstrates the stability of the Au_14_ (8e^–^) core. The Au_29_(S-Adm)_19_ structure, with a 10-electron core, which was solved by Li *et al.*,^26^ has explicitly revealed the correlation between the two Au_28_ and the fcc-Au_30_. Note that the Au_30_(S-Adm)_18_ (reported in ref (26)) and Au_30_(S-*t*Bu)_18_ have the same fcc core and a similar arrangement of staple motifs. The additional two electrons of Au_30_(SR)_18_ come from an added triangular Au_3_ unit on the bicuboctahedral Au_14_ core of Au_29_(SR)_19_. The series of Au_21_(SR)_15_ (6e^–^) to Au_36_(SR)_22_ (14e^–^) exhibits a uniform two-electron addition pattern in response to the core’s geometrical growth by adding Au_3_ and Au_4_ units (Figure 4).

### Theoretical Calculations

**Figure 5:**
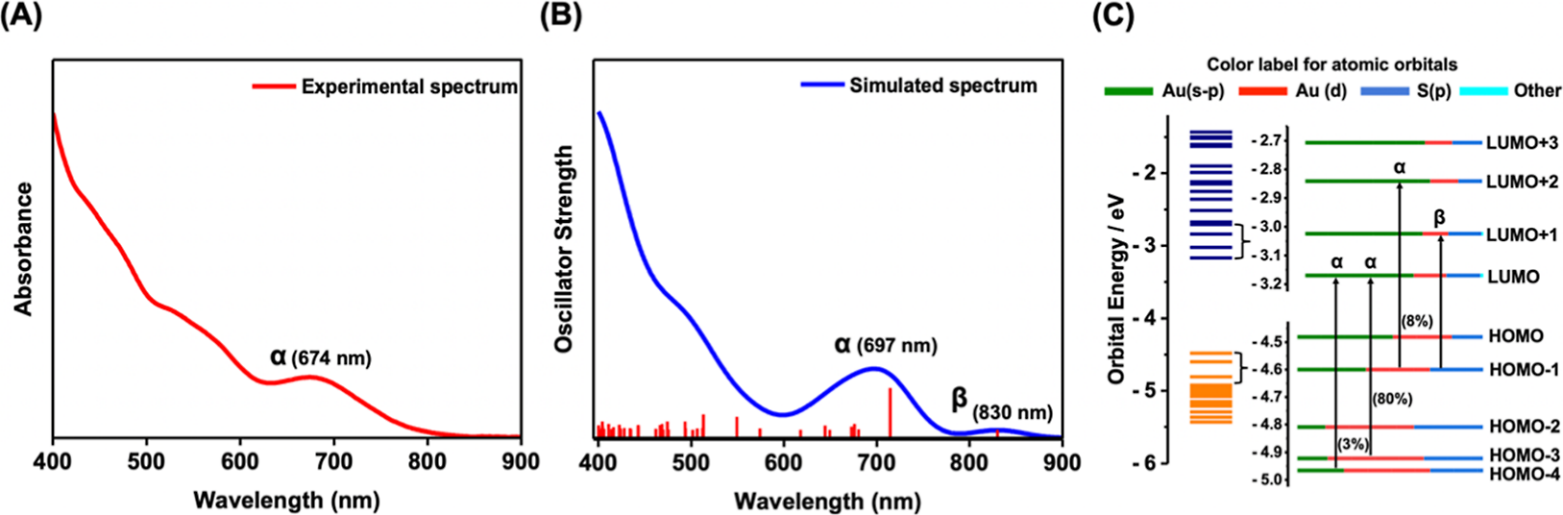
(A) Experimental absorption spectrum of Au_36_(S-*t*Bu)_22_. (B) TDDFT-simulated absorption spectrum of Au_36_(SCH_3_)_22_. (C) Kohn–Sham (KS) orbital energy level diagram of Au_36_(SCH_3_)_22_.

Time-dependent density functional theory (TDDFT) calculations were conducted in order to elucidate the electronic transitions involved in the optical absorption spectrum of Au_36_(S-*t*Bu)_22_. The structural data from the single-crystal X-ray diffraction was adopted as an input file to the quantum chemistry program Vienna Ab Initio Simulation Package (VASP). As shown in Figure 5A,B, the TDDFT-simulated absorption spectra match well with the experimental spectrum in the region of 400–800 nm. According to the calculated Kohn–Sham (KS) energy level diagram (Figure 5C), the strong absorption peak at 697 nm (denoted α) corresponds to the experimental peak at 674 nm and contains three transitions, namely, HOMO – 3 → LUMO (80%), HOMO – 1 → LUMO + 2 (8%), and HOMO – 4 → LUMO (3%). Both the HOMO and LUMO are mainly localized at the inner core and primarily composed of 6sp and 5d atomic orbitals of Au atoms. It is worth noting that the distribution of the electron density and nodes in the HOMO exhibit some features previously found in the core of some Au NCs constructed by Au_3_ and Au_4_ units^35^ (*i.e.*, each lobe is located in the corresponding Au_3_ or Au_4_ units of the core) (Figure S3). TDDFT simulations also showed a very weak peak (denoted as β) at 830 nm that is associated with the HOMO – 1 to LUMO + 1 transition, but this was not found in the experiment. To investigate whether the electronic transition for the 697 nm peak (the major one) is polarized, we computed its transition dipole moment. The components along the *x*, *y*, and *z* directions (defined in Figure S4) are *x* = 0.284, *y* = 0.154, and *z* = −0.134 Debye, where the – sign indicates that the *z* component (vector) is along the −*z* direction. One can see that the *x* direction’s value is ∼twice the *y* and *z* values; thus, the transition dipole is slightly polarized along the *x* direction due to the geometric structure.

### Thermo- and Photostability of Nanoclusters

**Figure 6:**
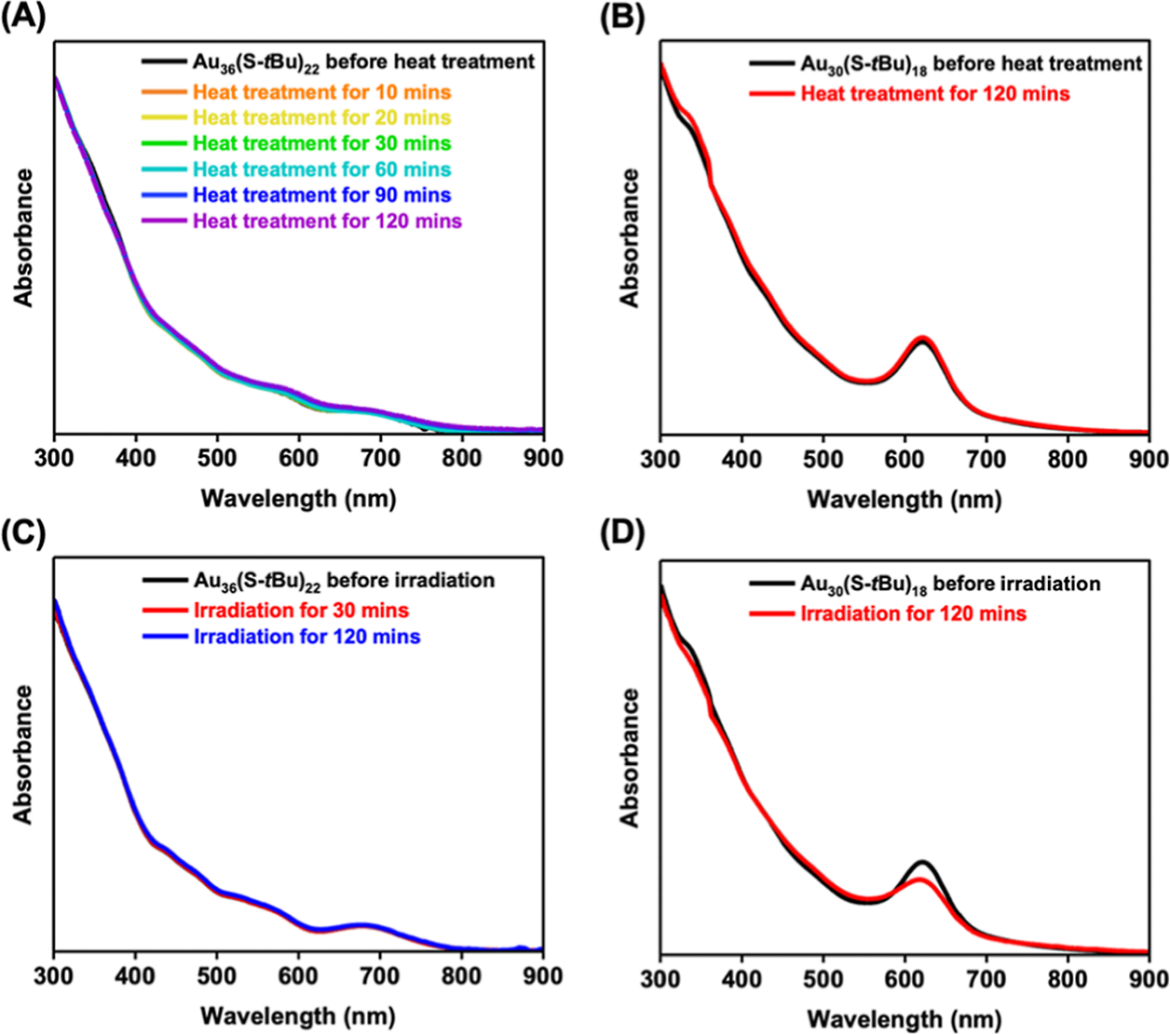
UV–vis absorption spectra of a toluene solution of Au_36_(S-*t*Bu)_22_ (A) and Au_30_(S-*t*Bu)_18_ (B) heated at 60 °C for 120 min. UV–vis absorption spectra of a toluene solution of Au_36_(S-*t*Bu)_22_ (C) and Au_30_(S-*t*Bu)_18_ (D) under irradiation at 365 nm for up to 120 min.

We further evaluated the thermo- and photostability of Au_36_(S-*t*Bu)_22_ and Au_30_(S-*t*Bu)_18_ by monitoring their UV–vis spectra. At 60 °C, both Au_36_(S-*t*Bu)_22_ and Au_30_(S-*t*Bu)_18_ show good stability in toluene for 120 min, evidenced by no discernible spectral changes (Figure 6A,B). Next, the photostability was evaluated by constant irradiation at 365 nm for NCs in toluene. We found that Au_36_(S-*t*Bu)_22_ is more stable than Au_30_(S-*t*Bu)_18_, as indicated by the constant spectra of Au_36_(S-*t*Bu)_22_, while Au_30_(S-*t*Bu)_18_ shows a decay of the 630 nm absorption peak (Figure 6C,D). From the structural point of view, Au_30_(S-*t*Bu)_18_ possesses a more symmetric Au_22_ core than the Au_25_ core of Au_36_(S-*t*Bu)_22_, and the symmetry would impart a higher stability, but this is not the case; we reason that the surface structures play a more important role. The presence of longer staple motifs in Au_36_(S-*t*Bu)_22_ should effectively protect the NC from the photoinduced degradation.^29^

## Conclusions

In summary, a Au_36_(S-*t*Bu)_22_ NC is synthesized and crystallized. X-ray analysis reveals that the Au_25_ core of Au_36_(S-*t*Bu)_22_ evolved from the Au_22_ core of Au_30_(S-*t*Bu)_18_ by adding a triangular Au_3_ fundamental unit, which follows the fcc structure evolutionary pattern of thiolate-protected Au_*n*_ NCs that are built up by cuboctahedral Au_13_ units. Compared to Au_30_(S-*t*Bu)_18_, the change in the core leads to surface motif differences in the Au_36_(S-*t*Bu)_22_. A five-fold improvement in PLQY of Au_36_(S-*t*Bu)_22_ over Au_30_(S-*t*Bu)_18_ is observed, which is due to the more than 10 times enhancement of the radiative rate constant in Au_36_(S-*t*Bu)_22_ due to the modification of its core. TDDFT calculations reveal the electronic transitions in the optical spectrum. The stability test indicates that the longer type of surface motif enhances the stability of Au_36_(S-*t*Bu)_22_ compared to that of Au_30_(S-*t*Bu)_18_. The results from this work provide important implications in correlating the structure and properties of cuboctahedral Au NCs as well as their geometric and electronic evolution. The findings, together with other types of structures such as icosahedral evolution,^7^ reveal fundamental insights into the geometric and electronic patterns for gold NCs^9,10,18,25^ and are expected to promote the development of their future applications.

## Methods

The synthesis of gold NCs was carried out by NaBH_4_ reduction of Au^I^–S-*t*Bu intermediates from thiol reduction of the gold salt. The target NCs were separated by thin-layer chromatography. Crystallization of NCs was performed by vapor diffusion of methanol into a toluene solution of NCs, followed by single-crystal X-ray diffraction analysis. Other characterization included optical absorption, PL (steady-state and time-resolved analyses), and theoretical simulations. More details can be found in the Supporting Information.

## Supporting Information Available

Experimental information including the synthetic details, lifetime measurements, single-crystal X-ray crystallography, details concerning computational methodology, and X-ray structure details (PDF)
Crystallography data of Au_36_(S-*t*Bu)_22_ (CIF)

## Supplementary Material

au4c00152_si_001.pdf

au4c00152_si_002.cif
