# Supplementary material for: Au36(SR)22 Nanocluster and a Periodic Pattern from Six to Fourteen Free Electrons in Core Size Evolution
Source: JACS Au. 2024 Apr 16;4(5):1928–34. doi: 10.1021/jacsau.4c00152 (PMC11134389; doi:10.1021/jacsau.4c00152)
Supplement: Supplementary file 1 — au4c00152_si_001.pdf [file au4c00152_si_001.pdf]

Supporting Information:

$Au_{36}(SR)_{22}$ Nanocluster and a Periodic Pattern from Six to Fourteen Free Electrons in Core Size Evolution

Yitong Wang[†$], Christopher G Gianopoulos[‡$], Zhongyu Liu[†$], Kristin Kirschbaum[‡], Dominic Alfonso[§*], Douglas R. Kauffman[§*], and Rongchao Jin[†*]

[†]Department of Chemistry, Carnegie Mellon University, Pittsburgh, Pennsylvania 15213, United States

[‡]Department of Chemistry and Biochemistry, University of Toledo, Toledo, Ohio 43606, United States

[§]National Energy Technology Laboratory, United States Department of Energy, Pittsburgh, Pennsylvania 15236, United States

[*]To whom correspondence should be addressed: Dominic.Alfonso@netl.doe.gov, Douglas.Kauffman@netl.doe.gov, rongchao@andrew.cmu.edu;

[$] Y.W., Z.L., C.G and D.A. contributed equally to this work.

Experimental:

1. Materials and Reagents

Tetrachloroauric (III) acid ($HAuCl_4 \cdot 3H_2O$, 99.99% metal basis, Aldrich), tert-butyl thiol (S-tBu, 98%, Aldrich), sodium borohydride ($NaBH_4$, Aldrich), tetrahydrofuran (HPLC grade, ≥99.9%, Aldrich), toluene (HPLC grade, ≥99.9%, Aldrich), dichloromethane (ACS reagent, ≥99.5%, Aldrich), acetonitrile (HPLC grade, ≥99.9%, Aldrich).

All chemicals were used without further purification. Nanopure water was prepared with a Barnstead NANOpure Diamond system. Thin-layer chromatography (TLC) plates were from iChromatography (silica gel, 250 μm).

2. Synthesis

$HAuCl_4 \cdot 3H_2O$ 98.74 mg (0.25 mmol) and 65.5 μL *tert*-butyl thiol were dissolved in 15 ml of THF under rapid stirring (550 rpm). The solution turned to deep orange in 30 minutes. At this point, a freshly prepared $NaBH_4$ solution (57 mg, 1.5 mmol) was rapidly added to the reaction mixture. Upon mixing, the solution immediately turned black. The reaction was allowed to proceed for three hours. Then, the reaction solution was evaporated to dryness. The black solid was washed three times with 50% aqueous methanol to remove excess thiol and extracted by 3 ml toluene. The vial containing Au NCs solution was then sealed and put in dark for incubation. After one week, the solution was concentrated and further purified by thin layer chromatography (TLC, developing solvent: hexane:dichloromethane = 3:2(v/v)). The yield of Au_{36}(S-*t*Bu)$_{22}$ is 6% based on gold atoms. The TLC separation of Au_{36}(S-*t*Bu)$_{22}$ is displayed in Figure S1 and the reproduciblilty of the synthesis of this compound is high. Of note, we found that the majority of NCs remained stable (judging by the optical absorption spectra) after 2 hours of etching with excess tert-butyl thiol at 60 ^{o}C, with a small amount transform into $Au_{30}(SR)_{18}$ and Au nanoparticles of larger sizes.

3. Steady-State UV-Vis-NIR Absorption Measurements

UV–vis–NIR spectra of all the Au NCs were collected on a UV-3600 Plus spectrophotometer (Shimadzu, range: 185-3300 nm).

4. Steady-State and Time-Resolved Photoluminescence Measurements

Steady-state photoluminescence (PL) spectra were measured on an FLS-1000 spectrofluorometer (Edinburgh). PL lifetimes were measured by time-correlated single photon counting (TCSPC) on the same instrument. Visible PL was measured using a photomultiplier (PMT) as the detector. Near-infrared PL was measured using a wide-range InGaAs detector (600-1600 nm) cooled to -80 °C with liquid nitrogen. The PLQY of Au NCs in toluene and DCM were determined by using $[Au_{25}(PPh_3)_{10}(SC_2H_4Ph)_5Cl_2]^{2+}$ nanocluster (PLQY: 8%)[1] as a reference.

5. Computational Methods

Nanocluster Model and Structural Optimization. The DFT calculations with the Perdew-Burke-Ernzerhof (PBE) functional and projector augmented wave (PAW) pseudopotential and projector augmented wave (PAW) pseudopotential were performed using the Vienna *Ab Initio* Simulation Package (VASP) 5.4.4.[2] The Kohn-Sham one electron valence eigenstates were expanded in terms of plane-wave basis sets with cutoff energy of 520 eV. The ionic and electronic convergence limit was set to 0.03 eV/Å and 1×10^{-5} eV, respectively, while the Methfessel-Paxton scheme was utilized with a smearing width of 0.2 eV.[3] The $Au_{36}(SR)_{22}$ (R=*tert*-butyl) nanocluster model is derived from the experimentally-solved crystal structures. Following previous works on atomically precise gold nanoclusters, the organic fragment of the ligands is modeled using a $-CH_3$ moiety in order to generate a computationally tractable model while accurately capturing the geometrical structure on the nanocluster.[4] The resulting 146-atom $Au_{36}(SCH_3)_{22}$ model was inserted into a three-dimensional 30 Å × 30 Å × 30 Å periodic cubic box to exclude periodic interaction between them. The sampling of the Brillouin zone was conducted with a Γ-point *k*-point mesh. The DFT calculations yield optimized geometries that are in very good agreement with experiment.

Electronic Structure and Photo-Absorption Spectra. In the present study, the time dependent-density functional theory (TDDFT) as implemented in TURBMOLE package (version 7.4)[5] was used to describe the electronic structure and photo-absorption spectra of $Au_{36}(SCH_3)_{22}$. The PBE functional together with the def2-SV(P) basis set[6] and respective effective core potentials to describe the inner electrons were chosen. Quadrature grids were of multiple grids m3 quality.[7] The resolution of identities approximation[8] were used to calculate 500 lowest singlet-to-singlet vertical energies. Simulated spectra were generated by convolving the calculated absorption energies and intensities with a Gaussian function of sigma set to 40 nm, and sampling the energy over 500 points within a 400-900 nm range.

6. X-ray Crystallography

A thin black plate of $Au_{36}(S\text{-}t\text{Bu})_{22}$ was used for data collection on a Bruker Duo diffractometer with a PHOTON II detector and Iμs CuKα radiation (1.54178 Å) at 200 K. The structure was solved in the triclinic space group $P\bar{1}$. Integration and scaling of the data yielded 104,900 reflections, of which 32,307 were unique (and 23,324 unique data with $I > 2\sigma(I)$), to a maximum of θ = 70.75° (d = 0.82 Å) with a completeness of 94.6% (99.5% out to θ = 50.00°; d= 1.00 Å) and an R_{int} of 10.34%.

The quality of numerous screened crystals was poor. Eventually, a single crystal of high quality was found and used for data collection. All Au and S atoms were refined with anisotropic displacement parameters, while all carbon atoms were treated with an isotropic displacement parameter. Two disordered lattice toluene molecules were identified and subjected to similarity restraint (SIMU) and the six phenyl carbon atoms constrained at the corners of a regular hexagon (AFIX 66), their occupancies were initially refined, and were constrained to 65% and 35% in the final refinement. The toluene methyl groups were

subjected to similar distance restraints (SADI) and restrained to be coplanar with the aromatic ring within 0.1 Å (FLAT). Hydrogen atoms were placed in idealized positions and treated with a riding model. Thus, the final model consisted of 907 parameters with 22 restraints.

The final refinement on F^2 converged at R_1 = 8.81% ($I > 2\sigma(I)$) and wR_2 = 27.68% (all data). The goodness-of-fit was 1.354. The largest residual density extrema were 7.41 and –3.39 ($e^-/Å^3$) with an RMS deviation of 0.57 $e^-/Å^3$. The highest two residual density maxima were located close to Au33 and Au31, a corresponding maximum was located in the vicinity of S2. Attempts were made to model this staple portion with a disorder model, occupancies refined to ~90% and 10%, but did not result in meaningful improvements to the model and the displacement parameters for the minor component were unusual when compared to other atoms in the model. Therefore, we have chosen not to include this potential disorder in our final model.

Supporting Figures:

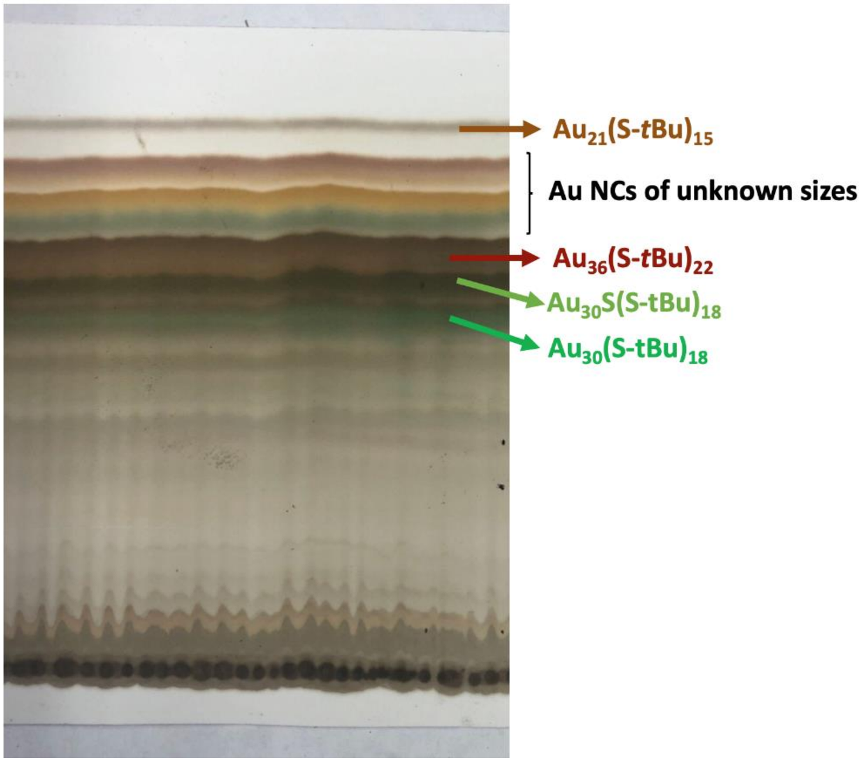

Figure S1. Thin-layer chromatography (TLC) separation of the crude products after incubation.

(a) **(b)**

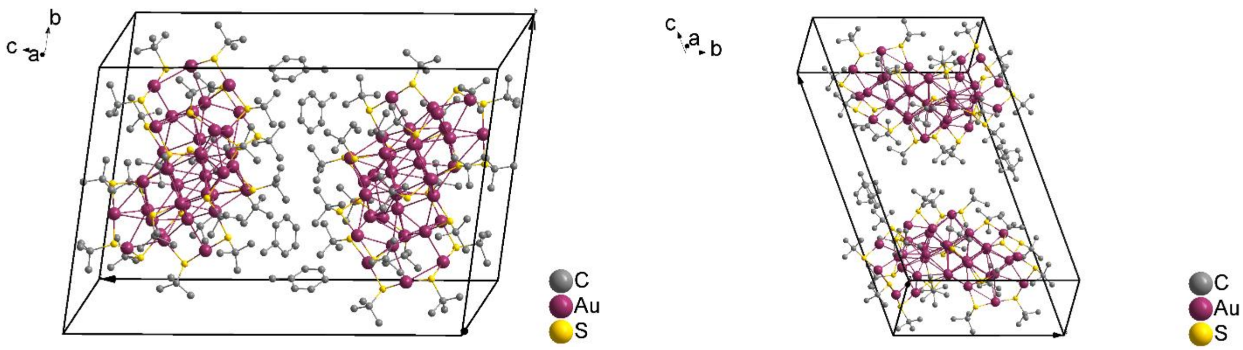

Figure S2. Ball-and-stick model of the experimental structure determined by single crystal X-ray crystallography for **Au_{36}(S-*t*Bu)$_{22}$**. The contents of one unit cell are depicted in two viewing directions (a and b) and H atoms have been omitted for clarity.

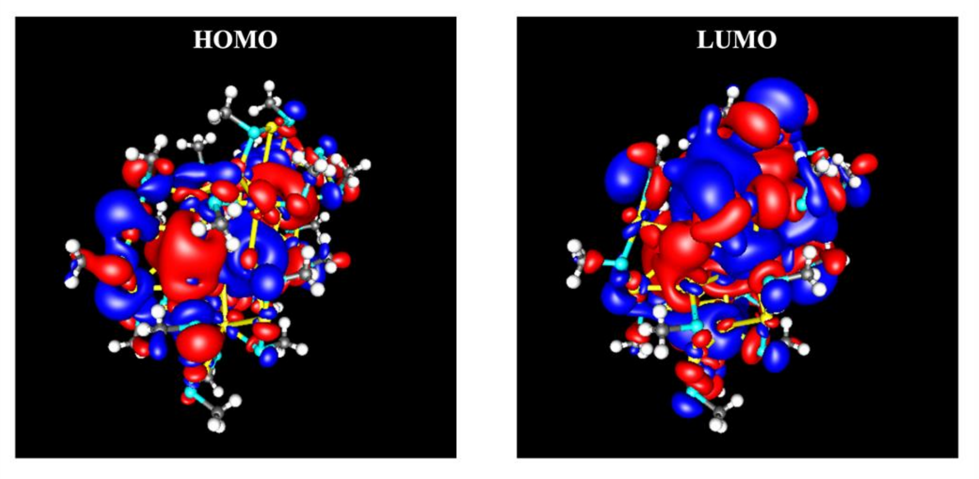

Figure S3. DFT-simulated HOMO and LUMO distribution in **Au_{36}(S-*t*Bu)$_{22}$**.

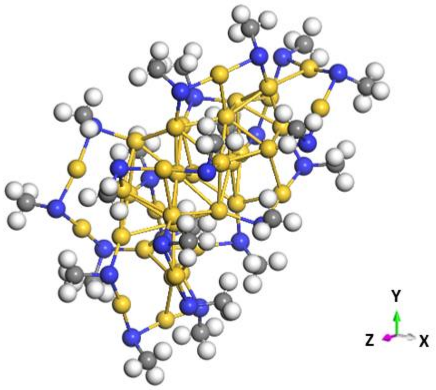

Figure S4. Computed transition dipole moment for the electronic transition involved in the major absorption peak of 697 nm. The x, y, and z components are x = 0.284, y = 0.154, z = –0.134 (the negative sign indicates the –z direction, see the figure).

Table S1. Sample and crystal data for $Au_{36}(S\text{-}tBu)_{22}$.

**Chemical formula**	$C_{88}H_{198}Au_{36}S_{22} \bullet C_7H_8$	
**Formula weight**	9144.70 g/mol	
**Temperature**	200(2) K	
**Wavelength**	1.54178 Å	
**Crystal size**	0.015 x 0.077 x 0.151 mm	
**Crystal system**	triclinic	
**Space group**	$P\bar{1}$	
**Unit cell dimensions**	a = 15.2750(5) Å	α = 96.4955(14)°
	b = 20.4443(6) Å	β = 90.6183(14)°
	c = 30.8045 Å	γ = 111.5528(14)°
**Volume**	8875.5(5) $Å^3$	
**Z**	2	
**Density (calculated)**	3.422 g/cm^3	
**Absorption coefficient**	56.930 mm^{-1}	
**F(000)**	7944	
**Theta range for data collection**	1.45° to 70.75°	
**Index ranges**	-17<=h<=18, -24<=k<=24, -37<=l<=35	
**Reflections collected**	104900	
**Independent reflections**	32307 [R(int) = 0.1027]	
**Coverage of independent reflections**	99.5% for theta = 50.00°	
**Absorption correction**	Multi-Scan	
**Max. and min. transmission**	0.9860 and 0.0082	
**Refinement method**	Full-matrix least-squares on F^2	
**Refinement program**	SHELXL-2019/2 (Sheldrick, 2019)	
**Function minimized**	$\Sigma w(F_o^2 - F_c^2)^2$	
**Data / restraints / parameters**	32307 / 22 / 907	
**Goodness-of-fit on F^2**	1.354	
**Final R indices[I>2σ(I)]**	R1 = 0.0881, wR2 = 0.2495	
**R indices (all data)**	R1 = 0.1167, wR2 = 0.2768	
**Largest diff. peak and hole**	7.408 and -3.393 $eÅ^{-3}$	
**R.M.S. deviation from mean**	0.566 $eÅ^{-3}$	

References:

(S1) Li, Q.; Zeman, C. J., IV; Ma, Z.; Schatz, G. C.; Gu, X. W. Bright NIR-II Photoluminescence in Rod-Shaped Icosahedral Gold Nano-clusters. *Small* **2021**, *17*, 200799.

(S2) (a) J. P. Perdew, K. Burke and M. Enzerhof. Generalized Gradient Approximation Made Simple. *Phys. Rev. Lett.*, 1996, **77**, 3865-3868; (b) Kresse, G.; Joubert, D. From ultrasoft pseudopotentials to the projector augmented-wave method. *Phys. Rev. B*, **1999**, 59, 1758-1775; (c) Kresse, G.; Furthmüller, J. Efficiency of ab-initio total energy calculations for metals and semiconductors using a plane-wave basis set. *Comp. Mat. Sci.*, **1996**, *6*, 15-50.

(S3) Methfessel, M.; Paxton, A. T. High-precision sampling for Brillouin-zone integration in metals. *Phys. Rev. B*, **1989**, *40*, 3616-3621.

(S4) (a) Jin, R.; Zeng, C.; Zhou, M.; Chen, Y. Atomically Precise Colloidal Metal Nanoclusters and Nanoparticles: Fundamentals and Opportunities. *Chem. Rev.* **2016**, *116*, 10346-10413; (b) Li, G.; Jin, R. Atomically Precise Gold Nanoclusters as New Model Catalysts. *Acc. Chem. Res.* **2013**, *46*, 1749-1758.

(S5) Ahlrichs, R.; Bär, M.; Häser, M.; Horn, H.; Kölmel, C. Electronic structure calculations on workstation computers: The program system turbomole. *Chem. Phys. Lett.* **1989**, *162*, 165-169.

(S6) Weigend, F.; Häser, M.; Patzelt, H.; Ahlrichs, R. Chem. RI-MP2: optimized auxiliary basis sets and demonstration of efficiency. *Phys. Lett.* **1998**, *294*, 143-152.

(S7) Treutler, O.; Ahlrichs, R. Efficient molecular numerical integration schemes. *J. Chem. Phys.* **1995**, *102*, 346.

(S8) Weigend, F.; Häser, M. RI-MP2: first derivatives and global consistency. *Theor. Chem. Acc*. **1997**, *97*, 331-340.
